# Human Cytomegalovirus Dysregulates Cellular Dual-Specificity Tyrosine Phosphorylation-Regulated Kinases and Sonic Hedgehog Pathway Proteins in Neural Astrocyte and Placental Models

**DOI:** 10.3390/v16060918

**Published:** 2024-06-05

**Authors:** Ece Egilmezer, Stuart T. Hamilton, Glen Lauw, Jasmine Follett, Eric Sonntag, Martin Schütz, Manfred Marschall, William D. Rawlinson

**Affiliations:** 1 Serology and Virology Division, Microbiology, NSW Health Pathology, Prince of Wales Hospital, Sydney 2031, Australia; ece.egilmezer1@health.nsw.gov.au (E.E.);; 2 School of Clinical Medicine, University of New South Wales, Kensington 2052, Australia; 3 School of Biotechnology and Biomolecular Sciences, University of New South Wales, Sydney 2033, Australia; 4 Institute for Clinical and Molecular Virology, Friedrich-Alexander University of Erlangen-Nürnberg, 91054 Erlangen, Germanymanfred.marschall@fau.de (M.M.)

**Keywords:** cytomegalovirus, pathogenesis, developmental pathways, astrocytes, placenta

## Abstract

Human cytomegalovirus (CMV) infection is the leading non-genetic cause of congenital malformation in developed countries, causing significant fetal injury, and in some cases fetal death. The pathogenetic mechanisms through which this host-specific virus infects then damages both the placenta and the fetal brain are currently ill-defined. We investigated the CMV modulation of key signaling pathway proteins for these organs including dual-specificity tyrosine phosphorylation-regulated kinases (DYRK) and Sonic Hedgehog (SHH) pathway proteins using human first trimester placental trophoblast (TEV-1) cells, primary human astrocyte (NHA) brain cells, and CMV-infected human placental tissue. Immunofluorescence demonstrated the accumulation and re-localization of SHH proteins in CMV-infected TEV-1 cells with Gli2, Ulk3, and Shh re-localizing to the CMV cytoplasmic virion assembly complex (VAC). In CMV-infected NHA cells, DYRK1A re-localized to the VAC and DYRK1B re-localized to the CMV nuclear replication compartments, and the SHH proteins re-localized with a similar pattern as was observed in TEV-1 cells. Western blot analysis in CMV-infected TEV-1 cells showed the upregulated expression of Rb, Ulk3, and Shh, but not Gli2. In CMV-infected NHA cells, there was an upregulation of DYRK1A, DYRK1B, Gli2, Rb, Ulk3, and Shh. These in vitro monoculture findings are consistent with patterns of protein upregulation and re-localization observed in naturally infected placental tissue and CMV-infected ex vivo placental explant histocultures. This study reveals CMV-induced changes in proteins critical for fetal development, and identifies new potential targets for CMV therapeutic development.

## 1. Introduction

Human cytomegalovirus (CMV) is the leading infectious cause of fetal malformation in developed countries [1]. The incidence of CMV births in developed countries ranges from 0.2 to 2.0%, and ranges from 0.6% to 6.1% in developing countries [1,2]. Congenital CMV infection can be asymptomatic, or result in fetal neural malformations including microcephaly, intracranial calcifications, cerebral palsy, mental disability, sensorineural hearing loss, seizures, and visual impairment [3,4,5]. The transplacental transmission of CMV from the infected placenta across the materno-fetal interface is the crucial and rate-limiting step for fetal infection [6]. In certain cases, infection may be restricted to the placenta instead, and there is evidence that placental CMV infection may indirectly result in adverse pregnancy outcomes [7,8,9]. The pathogenetic mechanisms of CMV-induced fetal and placental malformation have not yet been fully elucidated. 

Cellular protein kinases are dysregulated by CMV infection, and thought to be utilized by CMV to facilitate viral replication in a range of cells [10]. We have previously shown that dual-specificity tyrosine phosphorylation-regulated kinases (DYRKs) play a critical role in CMV replication in fibroblast cells, placental trophoblasts and placental ex vivo explant cultures [11]. The DYRK family of proteins are key regulators of cell growth, apoptosis, and differentiation, with DYRK1A and DYRK1B being the best characterized among the five mammalian DYRKs [12]. The DYRK1A protein is active in the nucleus and cytoplasm, and regulates cellular quiescence, and the proliferation and differentiation of neuronal progenitor cells, while also modulating signaling pathways including Sonic Hedgehog (SHH) [13,14,15,16,17]. DYRK1A is considered a significant gene in the Down syndrome phenotype and has been implicated in other neurological malformations, some of which are seen in congenital CMV infection [18]. These include intrauterine growth restriction (IUGR), developmental delay, and microcephaly [19,20,21]. DYRK1B, which has in comparison been less characterized, is associated with cellular differentiation, cell cycle arrest, and modulation of the SHH pathway [22,23]. 

One of the key functions of DYRK proteins is to modulate the SHH pathway, which is necessary for determining cell fate, patterning, proliferation, and differentiation [12]. In fetal brain development, the SHH pathway mediates midbrain and neural tube pattern formation, synapse formation, promotion of blood–brain barrier integrity, and cerebral immune quiescence mediated by astrocytes [24,25,26,27,28]. Recently, the significance of SHH in the placenta has been explored, showing the SHH pathway is required for placental development [29], and that the dysregulation of the pathway results in adverse pregnancy outcomes [30].

The effect CMV infection has on dysregulating critical fetal developmental pathways is emerging as a potential pathogenetic mechanism for fetal injury. Previously, we and others have shown the virus-supportive impact of DYRK activity on the cultured cell replication efficiency of CMV [11,31]. In this study, we utilized different in vitro and ex vivo cell culture systems to model (i) the placenta using first trimester extravillous trophoblast cells (TEV-1), placental explants, and clinically infected placental tissue to inform mother to child transmission (MTCT), and (ii) the fetal brain using normal human astrocytes (NHA). We show that CMV infection dysregulates DYRKs (DYRK1A, DYRK1B) and the SHH pathway (Shh morphogen, Gli2 transactivator, Ulk3 kinase, and Rb tumor suppressor protein). The CMV-induced dysregulation of these proteins that are vital for fetal development suggests an important viral pathogenetic mechanism for fetal neural and placental injury.

## 2. Methods

### 2.1. Cell Lines and Preparation of Virus Stocks

Human first-trimester extravillous trophoblast (TEV-1) cells were maintained in Ham’s F10 Nutrient Mix (Life Technologies, Carlsbad, CA, USA) supplemented with 10% fetal bovine serum (FBS; Interpath, Melbourne, VIC, Australia) and 100 U/mL penicillin G, 100 U/mL streptomycin and 29.2 µg/mL L-glutamine (1× PSG; Gibco, Sydney, NSW, Australia). Human NHA primary astrocytes (Lonza, Basel, Switzerland) were maintained in low-glucose Dulbecco’s modified Eagle medium (DMEM; Gibco) supplemented with 10% FBS and 1× PSG. Cell lines were mycoplasma-free and maintained at 37 °C with 5% CO_2_. 

The CMV strain AD169 (ATCC) was propagated in human MRC-5 cells maintained in MEM supplemented with 2% FBS and 1× PSG. The CMV strain Merlin (UL128+, RL13−) was propagated in RPE-1 cells as previously described [32]. Viral titres were determined using standard plaque assays. 

### 2.2. Clinical Placentae and Placental Villous Explant Histocultures

Clinical placental tissue was derived from formalin-fixed paraffin embedded (FFPE) placentae collected from still-born infants as part of a retrospective case series, as previously described [9]. The placenta with confirmed CMV infection was from a 21-week gestational age pregnancy and uninfected controls were matched for gestational age. 

Mock and Merlin-infected placental villous explant histocultures from term placenta were derived from FFPE explant sections from a previous study (under ethics approval SESIAHS HREC 09/126) as previously described [31,33]. 

### 2.3. Infection of TEV-1 Cells, and NHA Cells with CMV 

The TEV-1 and NHA cells were inoculated with CMV AD169 or CMV Merlin for Western blot analysis (0.5 pfu/cell for NHA and 2 pfu/cell for TEV-1), and inoculated with CMV AD169, CMV Merlin, or UV-inactivated CMV Merlin for immunofluorescence analysis (0.1 pfu/cell for NHA and 2 pfu/cell for TEV-1). Different multiplicities of infection for Western blot analysis were used to give approximately similar levels of infection, as determined by IFA between the different cell types. Lower MOIs were used for IFA experiments, to prevent all cells from being infected by 7 dpi, thereby allowing comparisons between CMV-infected and un-infected cells within the same experiment. Mock-infected cultures were established concurrently. Plates were centrifuged at 770× *g* for 30 min followed by 2 h incubation at 37 °C with 5% CO_2_, after which the medium was replaced with fresh cell culture media. Cells were incubated at 37 °C with 5% CO_2_ with cell culture media being replaced with fresh media at 4 dpi. 

### 2.4. Immunofluorescence

The TEV-1 and NHA cells were seeded in 6-well plates with underlying coverslips and infected with CMV AD169 and Merlin, as described above. Immunofluorescence staining was performed at days 1, 4, and 7 post-infection as previously described [34]. Formalin-fixed paraffin-embedded (FFPE) tissue sections of 4 µm were de-paraffinized and rehydrated followed by antigen retrieval using Tris-EDTA buffer (pH 9.0) for 20 min at 95 °C. These sections were incubated for one hour with primary antibodies for CMV detection with mouse mAb anti-HCMV immediate early (IE1p72) and early (pUL44) antibody cocktail (IE/E; clones DDG9 and CCH2; Dako, Sydney, NSW, Australia), mouse mAb-pp28 (Abcam, Cambridge, UK) and DYRK and Sonic Hedgehog (SHH) proteins with rabbit pAb-DYRK1A (Abcam), rabbit mAb-DYRK1B (Abcam), rabbit mAb-Sonic Hedgehog (Abcam), rabbit mAb-ULK3 (Abcam), rabbit mAb-Rb (Abcam) and rabbit pAb-Gli2 (Abcam). In our methods, we did not utilize an Fc blocking step as we have previously shown no differences in staining patterns observed with and without Fc blocking [31,34]. Sections and slides were incubated with secondary antibodies Alexa Fluor 488 goat anti-mouse and 594 goat donkey anti-rabbit (Invitrogen, Waltham, MA, USA; 1:1000 dilution) for 30 min. DAPI (Invitrogen) was added to each slide, mounted with coverslips and imaged as previously described [31]. 

As previously described, mock and Merlin-infected TEV-1 cells were stained with the non-specific control rabbit β-galactosidase (Abcam) antibody and PBS, to ensure staining was not due to non-specific primary and secondary antibody binding, respectively [31].

### 2.5. Western Blot Analysis

Western blot analysis was performed in duplicate using standard procedures, as described previously [35]. Immunostaining was performed using mAb anti-CMV immediate early (IE1p72) and early (pUL44) antibody cocktail (IE/E; clones DDG9 and CCH2; Dako), mAb-β-actin (Ac-15, Sigma, St. Louis, MO, USA), rAb-DYRK1A (Abcam), rAb-DYRK1B (Abcam), rAb-Shh (Abcam), rAb-ULK3 (Abcam), rAb-Rb (Abcam) and rAb-Gli2 (Abcam). HRP-conjugated anti-mouse or anti-rabbit secondary antibodies (Pierce) were used as secondary antibodies. Protein bands were visualized using chemiluminescence. Densitometry analysis was performed with ImageJ software (version 1.51j8) using experimental replicates and bands were normalized to β-actin expression. 

## 3. Results

### 3.1. CMV Infection of Trophoblast Cells Results in Accumulation and Re-Localization of SHH Proteins

**Figure 1:**
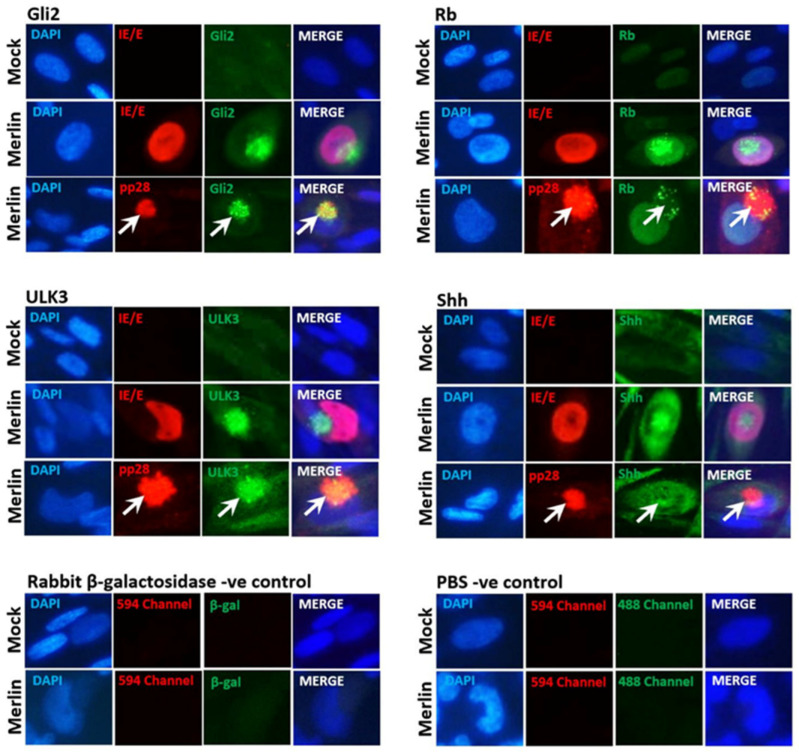
CMV infection of placental trophoblast cells results in the accumulation and re-localization of SHH proteins. TEV-1 trophoblast cells infected with CMV strain Merlin (2 pfu/cell) or mock-infected and stained for Gli2, Rb, Ulk3 or Shh protein and CMV immediate early IE1p72/early pUL44 (IE/E) or true late pp28 proteins at 7 dpi. The white arrows highlight the localization of pp28 staining. Mock- and Merlin-infected cultures incubated with non-specific rabbit mAb anti-β-galactosidase or PBS as primary antibody served as negative controls.

In a previous study, we showed that the AD169 and Merlin infection of TEV-1 cells resulted in cellular re-localization and accumulation of DYRK1A and DYRK1B [31]. In this study, immunofluorescence was performed on mock- and Merlin-infected TEV-1 cells at 7dpi for the Sonic Hedgehog signaling (SHH) proteins Gli2, Rb, Ulk3, and Shh (Figure 1). In mock-infected TEV-1 cells, Gli2, Ulk3, and Shh proteins were diffusely observed predominately in the cell cytoplasm. Merlin infection resulted in the accumulation of Gli2, Ulk3, and Shh in clusters of punctate staining in the cell cytoplasm. These proteins predominantly re-localized to the virion assembly complex (VAC), as demonstrated by co-localization with the VAC-associated CMV protein pp28 (UL99) (Figure 1). The Rb protein was observed in the nucleus of uninfected TEV-1 cells. Merlin infection induced the accumulation of Rb in the nucleus and partial re-localization to the VAC with punctate staining. 

The staining of cells with a non-specific rabbit β-galactosidase primary antibody or incubation with PBS did not result in any staining of the VAC, demonstrating the results were not due to non-specific CMV-derived Fc receptor binding.

To confirm that the changes observed in protein localization were due to CMV replication rather than a cellular stress response or virus-cell binding/entry, TEV-1 cells were inoculated with UV-inactivated Merlin. No changes were observed in the localization or accumulation of SHH proteins relative to mock-infected TEV-1 cells (Supplementary Figure S1). 

### 3.2. CMV-Induced Trophoblast Accumulation and Re-Localization of Rb, Ulk3, and Shh, but Not Gli2 Is Caused by Upregulation of Expression

**Figure 2:**
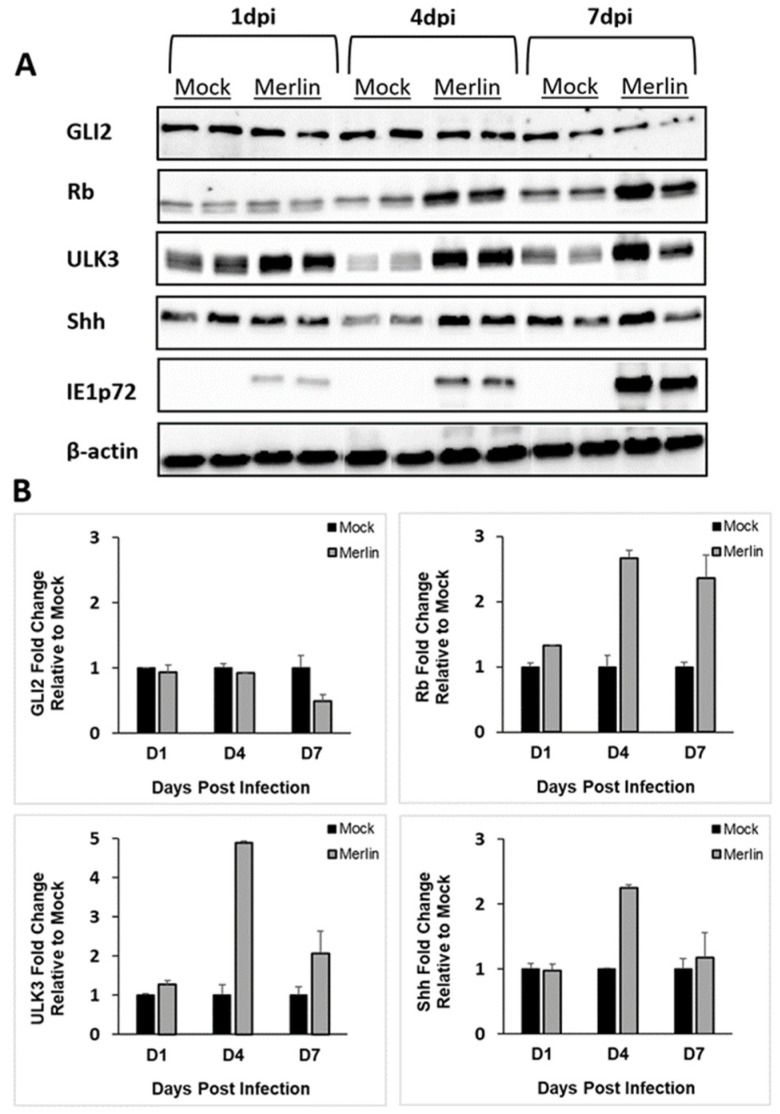
CMV infection of trophoblast cells results in increased protein expression of key SHH proteins. (**A**) TEV-1 trophoblast cells were infected with CMV strain Merlin (2 pfu/cell) and cells harvested at 1, 4 and 7 dpi with cellular Gli2, Rb, Ulk3, Shh or CMV immediate early IE1p72 protein expression measured using Western blot. (**B**) Gli2, Rb, Ulk3 or Shh protein expression fold change, normalized to β-actin levels in Merlin-infected cells relative to mock-infected cells using densitometry. Results derived from duplicate experiments and densitometry results presented as mean ± SD.

Western blot analyses were performed to determine the effects of CMV-infection on the expression of SHH proteins in TEV-1 cells (Figure 2A). Densitometry analysis using ImageJ was performed to quantitate protein expression in CMV-infected cells relative to the uninfected (mock) control cells at days 1, 4, and 7 post-infection, and the bands were normalized to β-actin (Figure 2B). At 1 dpi, no alterations were observed in Gli2, Rb, Ulk3, or Shh levels in Merlin-infected cells relative to mock. At 4 dpi, Rb, Ulk3, and Shh protein levels increased, but not Gli2 in Merlin-infected TEV-1 cells relative to mock. At 7dpi, relative to mock-infected cells, Rb, Ulk3, and Shh protein expressions increased, and Gli2 exhibited a small reduction in protein levels in Merlin-infected cells.

### 3.3. CMV Infection of Normal Human Astrocyte Cells Results in Accumulation and Re-Localization of DYRK and SHH Proteins 

**Figure 3:**
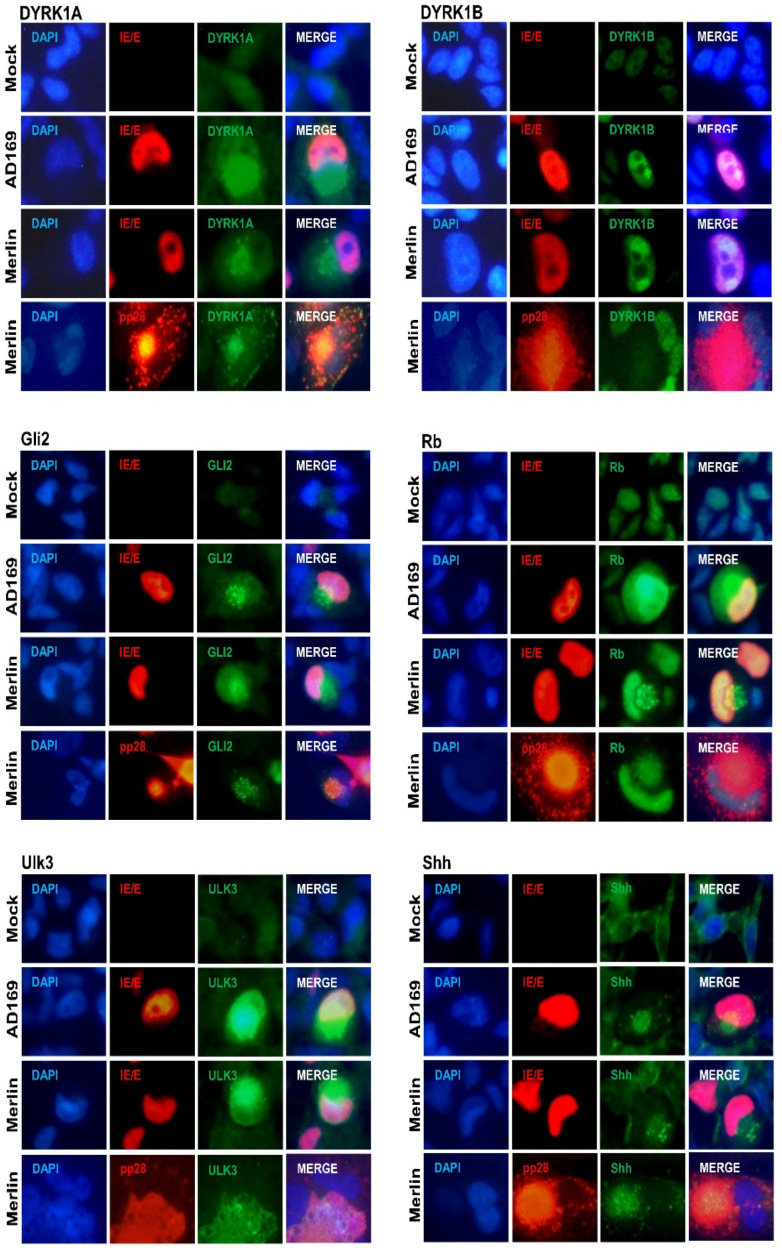
CMV infection of human brain astrocyte cells results in the similar accumulation and re-localization of SHH proteins as placental trophoblast cells. NHA astrocyte cells infected with CMV AD169 or Merlin strain (0.1 pfu/cell) or mock-infected and stained for DYRK1A, DYRK1B, Gli2, Rb, Ulk3 or Shh protein and CMV immediate early IE1p72/early pUL44 (IE/E) and true late pp28 (UL99) proteins at 7 dpi.

We have previously shown that the CMV infection of placental trophoblasts, ex vivo placental explants, and naturally infected clinical placentae results in the upregulation and re-localization of DYRK1A to the cytoplasm, and VAC and DYRK1B to the nucleus [31]. Immunofluorescence was performed at 7 dpi on mock-, AD169-, and Merlin-infected NHA cells for DYRK and SHH proteins (Figure 3). In mock-infected NHAs, DYRK1A, Gli2, and Ulk3 were diffusely localized in the nucleus and cytoplasm. The DYRK1B and Rb proteins were diffusely localized in the nucleus, and Shh in the cytoplasm. Upon AD169 or Merlin infection, the aberrant re-localization and accumulation of these proteins were detected. The DYRK1A, Gli2, and Shh proteins accumulated in the cytoplasmic virion assembly complex. The small punctate accumulation of DYRK1B was observed in infected cell nuclei corresponding to the CMV nuclear replication compartments. Rb and Ulk3 accumulated in both the nucleus and cytoplasm of infected cells relative to mock.

No differences in re-localization patterns were observed between AD169- and Merlin-infected NHA cells. The CMV-induced accumulation and localization patterns for the SHH proteins were comparable to patterns observed in CMV-infected placental trophoblast cells (Figure 1). 

Merlin-infected NHA cells were additionally co-stained for the DYRK (DYRK1A and DYRK1B) and SHH (Gli2, Rb, Ulk3, and Shh) proteins with the true late CMV protein pp28. Similar to TEV-1 cells (Figure 1), these proteins predominantly re-localized to the VAC (Figure 3). The inoculation of NHA cells with UV-inactivated Merlin showed no changes in the localization or accumulation of DYRK or SHH proteins relative to mock-infected NHA cells (Supplementary Figure S1). 

### 3.4. CMV-Induced Normal Human Astrocyte Accumulation and Re-Localization of DYRK and SHH Proteins Is Caused by Upregulated Expression

**Figure 4:**
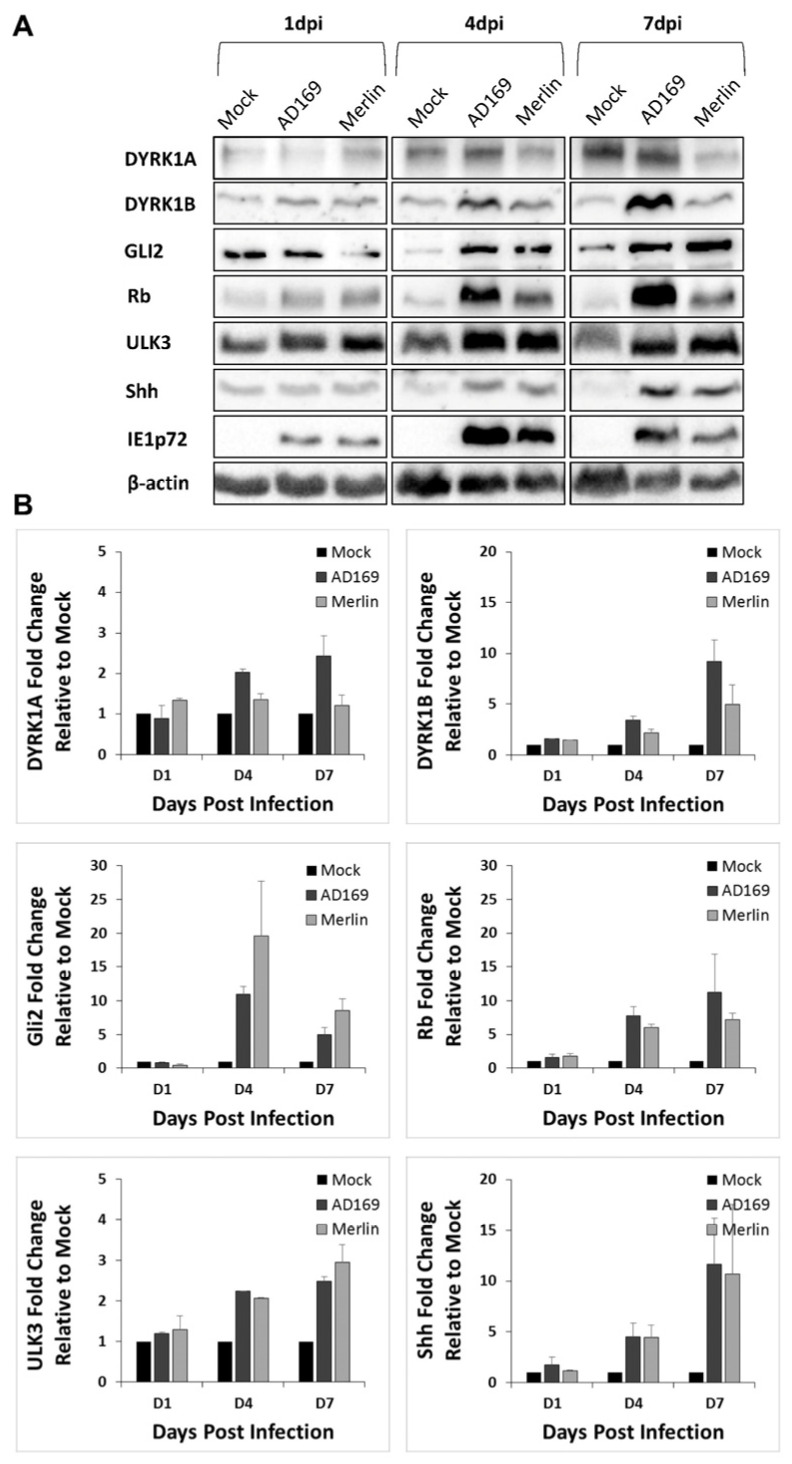
CMV infection of human brain astrocyte cells results in increased protein expression of key SHH proteins. (**A**) NHA astrocyte cells were infected with CMV strain AD169 or Merlin (0.5 pfu/cell) and cells were harvested at 1, 4 and 7 dpi, with cellular DYRK1A, DYRK1B, Gli2, Rb, Ulk3, Shh or CMV immediate early IE1p72 protein expression measured using Western blot. (**B**) DYRK1A, DYRK1B, Gli2, Rb, Ulk3 or Shh protein expression fold change, normalized to β-actin levels in Merlin-infected cells relative to mock-infected cells using densitometry. Results derived from duplicate experiments and densitometry results presented as mean ± SD.

Western blot analyses were performed to determine the effects of CMV-infection on the expression of DYRK and SHH proteins in NHA cells (Figure 4). Densitometry analysis using ImageJ was performed to quantitate protein expression in CMV-infected cells relative to the uninfected (mock) control cells at days 1, 4, and 7 post-infection, and the bands were normalized to β-actin. At 7 dpi, DYRK1A was only upregulated in AD169-infected cells but not in Merlin-infected cells, which may be a biological consequence of the difference between CMV strains. Upregulation was detected in DYRK1B, Gli2, Rb, Ulk3, and Shh in both AD169- and Merlin-infected NHA cells. The changes in protein expression in CMV-infected NHA cells were time-dependent, and these proteins were dysregulated during early to late stages of CMV replication, i.e., 4 dpi and 7 dpi.

### 3.5. The CMV Upregulation of SHH Proteins Occurs in In Vivo and Ex Vivo Human Placental Models

**Figure 5:**
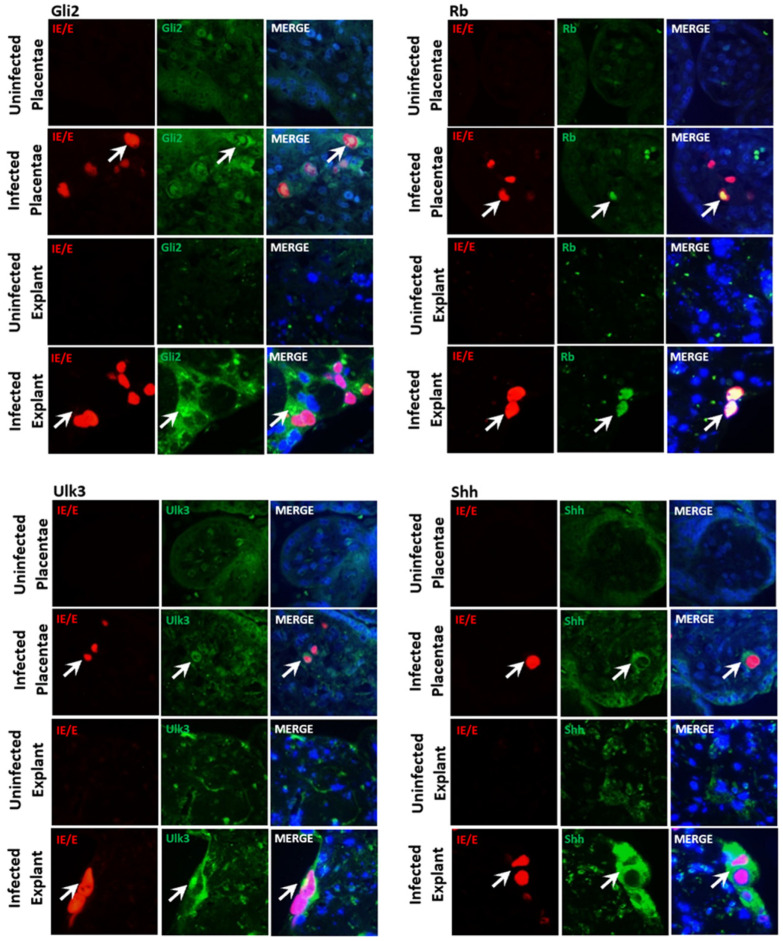
CMV infection in naturally infected and Merlin-infected placental tissue results in the upregulation and re-localization of key SHH proteins. Naturally infected clinical placentae, Merlin-infected ex vivo placental explants or uninfected clinical placentae and placental explants stained for CMV immediate early/early protein (IE/E) and Gli2, Rb, Ulk3 and Shh proteins. Representative images of clinical placentae derived from 22-week gestational age placental tissue matched for gestational age control or placental villous explant histocultures from term placentae. The white arrows indicate infected cells of interest in each panel.

Expressions of the SHH proteins Gli2, Rb, Ulk3, and Shh were investigated in multicellular human placentae infected with CMV both naturally and ex vivo to complement the evidence in cell culture monolayers, as we reported previously for DYRK1A and DYRK1B [31]. Both Gli2 and Rb were faintly expressed in cells composing the placental villi in both CMV-infected and uninfected placentae (Figure 5). In CMV-infected placental sections, cytoplasmic and some nuclear re-localization of Gli2 was observed in CMV-infected cells. Rb staining in CMV-infected cells showed increased presence in the nucleus compared to uninfected placental cells, with no signs of punctate staining, as was observed in cell monocultures (Figure 5). Ulk3 was primarily concentrated in the cytoplasm of cells within the villi of CMV-infected and uninfected placentae, with a modest increase in cytoplasmic staining in CMV-infected cells in infected tissue. Shh expression was detected primarily in the cytoplasm of cells within the villi, again with increased cytoplasmic staining in CMV-infected cells of the placental tissues. 

## 4. Discussion

Congenital cytomegalovirus pathogenesis likely involves cellular dysregulation in placental and fetal compartments, both of which likely contribute to fetal damage [36]. We have previously shown DYRKs play a critical role in CMV replication in in vitro fibroblasts, placental trophoblasts, and placental ex vivo explant cultures, as well as the dysregulation of these proteins in naturally infected clinical placental tissue [11,31]. Protein kinases play a critical role in the regulation of cellular functions through signal transduction cascades [37], and as a result, the dysregulation of these kinases represents an interesting pathogenetic mechanism for fetal neural malformation. 

The importance of the DYRK-influenced SHH pathway to the development of placentae is an emerging area of interest. The Shh and Gli2 proteins are required for appropriate placental development and pregnancy maintenance in murine models [29]. A study recently reported the dysregulation of the SHH pathway in preeclampsia placentae samples [30]. In this study, we showed that the Merlin infection of human first trimester extravillous trophoblast (TEV-1) cells increased Rb, Ulk3, and Shh protein levels relative to uninfected (mock) controls. Evidence suggests that a reduction in Ulk3 mRNA levels in Shh-responsive cells increases the cells’ potency to transmit the Shh signal [38]. Similarly, Rb has been shown to exhibit a negative correlation with the SHH pathway [39]. Taken together, the increase in Ulk3 and Rb in CMV-infected TEV-1 cells suggests the suppression of the SHH pathway in infected placentae. In primary cytotrophoblasts, it has been reported that trophoblast syncytialization is modulated by non-canonical SHH signaling [30]. Cytotrophoblasts undergo syncitialization during placental development to form the syncytiotrophoblast layer, which is essential in transporting almost all nutrients from the mother to the fetus [40,41]. Similarly, the infection of cytotrophoblasts with CMV has also been shown to suppress syncytialization [42]. Changes in the rates of syncytialization have been observed in several placentae pathologies, including intrauterine growth restriction (IUGR) or miscarriage [43], both of which are associated sequelae of CMV infection [44]. The dysregulation of SHH signaling in trophoblasts during CMV infection may therefore be a potential mechanism for aberrant syncitialization, resulting in the adverse pregnancy outcomes observed in cases of congenital CMV infection. 

DYRKs are regulators of the SHH pathway, which is critical during embryogenesis and fetal brain development [16,24,25,45]. We have previously shown DYRK proteins play a critical role in CMV replication in fibroblasts, placental trophoblasts, and three-dimensional placental ex vivo explant cultures [11,31]. We have also shown the upregulation of Ulk3, Gli2, and Rb in CMV-infected HFF cells [11]. Here, we show CMV-induced dysregulation of the critical placental and neuro-developmental pathways, DYRK (DYRK1A, DYRK1B) and SHH (Gli2, Rb, Ulk3, and Shh), utilizing NHA cells to model the CMV infection of human astrocytes. Astrocytes are the most abundant cell type in the brain. They have critical functions including modulating the cerebral immune response, providing structural support for neurons, modulating synaptic activity and plasticity, and regulating neurotransmitters [46,47,48,49]. Human astrocytes are susceptible to CMV infection [50,51]. Here, we show that both AD169 and Merlin strain-infected NHA cells exhibited increased DYRK1A and DYRK1B protein levels relative to mock. The DYRK1A gene, which is considerably better characterized compared to DYRK1B, is significantly associated with the Down syndrome phenotype [18]. The over-expression of DYRK1A is a suggested mechanism for inducing the neurofibrillary degeneration in Down syndrome individuals through tau hyperphoshorylation [52]. In a study with 14 individuals with de novo heterozygous variants of DYRK1A, all were reported to have congenital microcephaly, intellectual disability, developmental delay, and speech impairments, all of which are also sequelae of congenital CMV infection [18]. This suggests a possible link between the CMV-induced dysregulation of DYRK proteins, and the development of neurological sequelae. These data, combined with the findings in this study, show that the CMV infection of NHAs dysregulates DYRK protein levels and localization, suggesting a mechanism of CMV-induced fetal neural injury. The differential re-localization of DYRK1A and DYRK1B observed in CMV-infected NHA cells, with similar re-localization patterns as were previously observed in placental models, suggests CMV dysregulates the two protein kinases in separate processes during viral replication [31]. 

The SHH pathway, which is modulated by DYRKs, is essential for fetal development, facilitating blood–brain barrier integrity, cerebral immune quiescence mediated by astrocytes, and axon pathfinding [24,25,26,27,28,53]. The SHH signaling pathway plays a significant role in astrocyte function, particularly the crosstalk between astrocytes and neurons [53]. In NHA cells, CMV infection induced an increase and re-localization in all SHH proteins by 7dpi. Recently, it has been reported that astrocyte-specific SHH pathway activation is necessary for synapse formation [54], and the selective disruption of SHH in astrocytes substantially increases synapse numbers [27]. Synapse number and distribution is critically regulated during brain development, and failure to establish an appropriate organization of synapses is the hallmark of a number of neurodevelopmental disorders such as autism [27,55]. Future experiments could benefit from the use of confocal microscopy due to its ability to examine the three-dimensional aspect of culture samples. This would facilitate further analysis of the CMV-induced co-localization patterns of DYRK and SHH proteins. The dysregulation of SHH proteins in NHA cells reported in this study suggests a possible mechanism for the development of neurodevelopmental disease. 

This study revealed the dysregulation of cellular kinases (DYRKs) and proteins from the key developmental pathway, SHH, in CMV-infected placental and brain models. The CMV-induced dysregulation of protein levels and re-localization in the placental and neural models is indicative of potential viral pathogenetic mechanisms. These data can be further utilized to investigate CMV-induced adverse pregnancy outcomes and novel therapeutic discovery in preventing the dysregulated signaling pathways of the placenta and fetus [9,35,56]. 

## Data Availability

The authors confirm that the data supporting the findings of this study are available within the article and Supplementary Materials.

## Supplementary Materials

The following supporting information can be downloaded at: https://www.mdpi.com/article/10.3390/v16060918/s1.
