# Supplementary material for: Human Cytomegalovirus Dysregulates Cellular Dual-Specificity Tyrosine Phosphorylation-Regulated Kinases and Sonic Hedgehog Pathway Proteins in Neural Astrocyte and Placental Models
Source: Viruses. 2024 Jun 5;16(6):918. doi: 10.3390/v16060918 (PMC11209403; doi:10.3390/v16060918)
Supplement: Supplementary file 1 [file viruses-16-00918-s001.zip › viruses-2845532-supplementary.pdf]

Scheme S1. UV-inactivated Merlin inoculation of TEV-1 and NHA cells yields similar localisation of key DYRK and SHH proteins relative to mock. Immunofluorescence was performed on TEV-1 and NHA cells inoculated with UV-inactivated Merlin at 7dpi. Similar localisation patterns of key DYRK and SHH proteins relative to mock-infected cells indicates the observed re-localisation effects of CMV infection are a result of CMV replication.

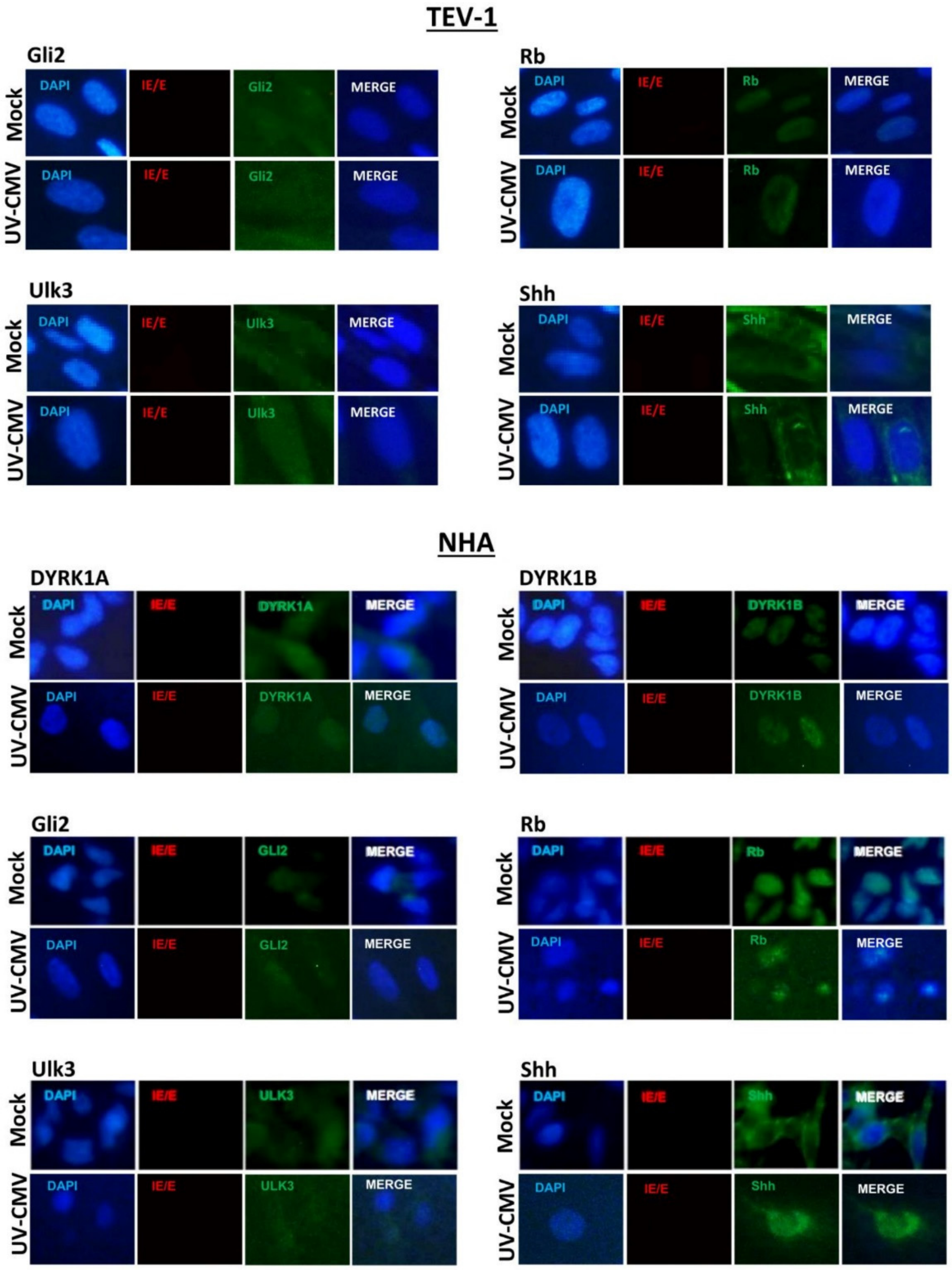

Supplementary Figure S1

Supplementary Data
